# Global transcriptome analysis of developing chickpea (*Cicer arietinum* L.) seeds

**DOI:** 10.3389/fpls.2014.00698

**Published:** 2014-12-16

**Authors:** Seema Pradhan, Nitesh Bandhiwal, Niraj Shah, Chandra Kant, Rashmi Gaur, Sabhyata Bhatia

**Affiliations:** National Institute of Plant Genome ResearchNew Delhi, India

**Keywords:** Transcriptome, chickpea, seed, assembly, next generation sequencing, annotation, differential expression

## Abstract

Understanding developmental processes, especially in non-model crop plants, is extremely important in order to unravel unique mechanisms regulating development. Chickpea (*C. arietinum* L.) seeds are especially valued for their high carbohydrate and protein content. Therefore, in order to elucidate the mechanisms underlying seed development in chickpea, deep sequencing of transcriptomes from four developmental stages was undertaken. In this study, next generation sequencing platform was utilized to sequence the transcriptome of four distinct stages of seed development in chickpea. About 1.3 million reads were generated which were assembled into 51,099 unigenes by merging the *de novo* and reference assemblies. Functional annotation of the unigenes was carried out using the Uniprot, COG and KEGG databases. RPKM based digital expression analysis revealed specific gene activities at different stages of development which was validated using Real time PCR analysis. More than 90% of the unigenes were found to be expressed in at least one of the four seed tissues. DEGseq was used to determine differentially expressing genes which revealed that only 6.75% of the unigenes were differentially expressed at various stages. Homology based comparison revealed 17.5% of the unigenes to be putatively seed specific. Transcription factors were predicted based on HMM profiles built using TF sequences from five legume plants and analyzed for their differential expression during progression of seed development. Expression analysis of genes involved in biosynthesis of important secondary metabolites suggested that chickpea seeds can serve as a good source of antioxidants. Since transcriptomes are a valuable source of molecular markers like simple sequence repeats (SSRs), about 12,000 SSRs were mined in chickpea seed transcriptome and few of them were validated. In conclusion, this study will serve as a valuable resource for improved chickpea breeding.

## Introduction

Chickpea (*Cicer arietinum* L.) is one of the earliest annual pulse crops to be cultivated by man and consumed as a source of vegetable protein and the third most important food legume in the world. India is the leading producer of chickpea where the crop is grown on 8.3 million hectares of land yielding 7.7 million tons of chickpea crop. Of these, 0.33 million tons comprises the seeds (FAOSTAT, 2012^1^, http://faostat3.fao.org/faostat-gateway/go/to/download/Q/QC/E). Chickpea is a self-pollinating diploid (2*n* = 16) annual crop with a genome size of 738.09 Mbp (Varshney et al., 2013). The seeds are an important source of protein for millions of people in the developing countries. In addition to having high protein content (20–23%), chickpea seeds are rich in carbohydrates (41.1–47.42%), fiber (6% crude fiber), minerals (phosphorus, calcium, magnesium, iron and zinc) and β-carotene (Friedman, 1996; Abbo et al., 2005; Iqbal et al., 2006). Apart from all the obvious dietary attributes, improving the yield and quality of chickpeas is economically desirable keeping in mind the large population of developing countries that are in need of an affordable source of high protein nutrition. Seeds are essential for flowering plant reproduction because they protect, nourish and contain the developing embryo that represents the next sporophytic generation. Various studies on legume seed development show that the process is genetically programmed and correlated with changes in metabolite levels (Borisjuk et al., 2004; Weber et al., 2005).

In order to explore the possibilities of improvement in the nutritional quality and quantity of the seeds, it is necessary to have a clear understanding of the regulatory mechanisms involved in the process of seed development. Such an understanding may be best achieved through an exhaustive study of the transcriptome of the developing seed which may provide a blueprint of genes and pathways involved. Conventional methods for such studies have included generation of EST libraries by cloning and sequencing on the Sanger platform and have been used for analyzing the transcriptome of developing seeds in plants such as *Arabidopsis thaliana* (White et al., 2000). But the advent of high throughput NGS technology enables us to generate considerably larger amounts of data as compared to conventional transcript analysis methods. A host of such technologies from Illumina/Solexa, ABI/SOLiD and 454/Roche have provided unprecedented opportunities for high-throughput functional genomics research (Mardis, 2008; Schuster, 2008; Morozova et al., 2009). The efficiency of the Roche/454 Genome Sequencer FLX platform for improving the coverage of transcriptome as compared to conventional methods of EST sequencing has been well documented (Cheung et al., 2006). Also, many transcriptome sequencing projects have preferred to use the 454 platform as compared to other techniques (Vera et al., 2008; Salem et al., 2010) due to longer reads (~600–800 bp) which are thought to be more amenable to *de novo* assembly and annotation. Earlier studies related to in depth analysis of seed development have been undertaken using various high throughput techniques in plants like *Arabidopsis* (Girke et al., 2000; Le et al., 2010), wheat (Gregersen et al., 2005) rice (Xue et al., 2012) and oats (Gutierrez-Gonzalez et al., 2013) and also in important legumes such as soybean (Severin et al., 2010; Jones and Vodkin, 2013) and *Medicago* (Gallardo et al., 2007).

Although there have been studies reporting transcriptome analysis in chickpea (Hiremath et al., 2011; Garg et al., 2011a,b; Singh et al., 2013; Afonso-Grunz et al., 2014; Kudapa et al., 2014), none of these have focussed specifically on chickpea seed development. However, such studies have been undertaken in the related legume soybean where seed development was extensively studied with respect to different tissues (Severin et al., 2010) as well as in seeds taking developmental stages from few days post fertilization to maturity where major landmarks in seed development, including accumulation of nutrients, synthesis of storage proteins and desiccation were analyzed using RNA-seq (Jones and Vodkin, 2013).

Seed development in dicots generally proceeds through three stages. The first stage is that of morphological development where embryo formation occurs. In the second stage, i.e., cell expansion, food and storage reserves are produced while in the third stage, i.e., maturation drying, desiccation of seed occurs (Baud et al., 2002). Earlier studies on chickpea seed development (Setia and Malik, 1985) have shown that an exponential increase in fresh seed weight occurs between 10 and 40 days after anthesis (DAA) which was also observed in our chickpea samples (inset Table 1). Hence, we selected the following time points: 10 DAA, to correlate with early stages (cell division and morphogenesis); 20 DAA and 30 DAA, to correlate with middle stages (cell expansion and storage protein accumulation) and 40 DAA to correlate with maturation, thereby representing the complete spectrum of seed development from early stages to maturity. Further, we compared the transcripts obtained from each stage with those from other tissues of chickpea to find out genes specific to chickpea seed development. Digital expression analysis of the transcripts from individual stages was carried out to provide information about developmental stage specific events. Identification of the transcription factors and metabolic pathways involved in seed development was done in order to gain an insight into the regulatory networks governing seed development.

**Table 1:**
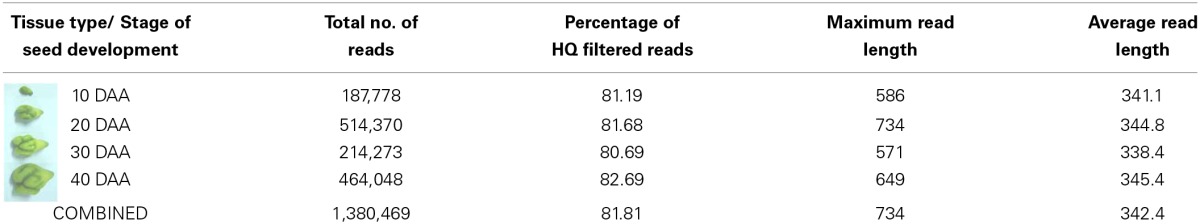
**Summary of sequenced reads obtained from the four stages of developing seed tissues**.

## Materials and methods

### Plant materials

Chickpea (*C. arietinum*) cultivar ICCV2 was grown in the field at NIPGR. The flowers were tagged on the day they opened completely. Seeds were collected at 10, 20, 30, and 40 DAA intervals, frozen in liquid nitrogen and stored at −80°C. At least three biological replicates were collected for each sample.

### RNA isolation and cDNA synthesis

Total RNA was isolated from tissue samples (pooled from three biological replicates) using the LiCl precipitation method as described by Choudhary et al. (2009). Briefly, 0.8g of frozen seed tissue was ground to a fine powder in liquid nitrogen and transferred to an Eppendorf tube containing 500 μl of extraction buffer (200 mM NaOAc pH-5.2, 1% SDS, 10 mM EDTA pH 8.0) and 500 μl of phenol. This was centrifuged at 14,000g for 10 min at RT. The aqueous phase was separated and extracted twice with phenol:chloroform (1:1) followed by O/N precipitation at 4°C with 0.3 volume of 10 M LiCl for RNA precipitation. The RNA pellet was recovered by centrifugation at 10,000 rpm/9700g for 10 min at 4°C, and was washed twice with 2.5 M LiCl and once with 70% ethanol. The pellet was air-dried and dissolved in DEPC-treated ddH2O. mRNA was isolated from the total RNA using PolyATract mRNA isolation system IV (Promega Corporation, Madison, WI, USA) according to the manufacturer's protocol. Both total RNA and mRNA samples were checked for quality on the Agilent 2100 Bioanalyzer (Agilent Technologies, California, USA) and only samples having RIN (RNA Integrity Number) greater than 8.0 were selected for cDNA preparation. cDNA was synthesized from 1.5 μg of mRNA using Promega's Universal Riboclone cDNA synthesis system (Promega Corporation, Madison, WI, USA) using random hexameric primers and following the manufacturer's protocol.

### 454 (GSFLX Titanium) sequencing

Approximately 600ng of double-stranded cDNA was nebulized and fragments ranging in size from 300 to 800 bp were selected. Specific adapters were ligated to the fragmented cDNA and denatured to generate single-stranded cDNA followed by emulsion PCR amplification for sequencing. Four cDNA libraries were generated, one each from the mRNA isolated from 10, 20, 30, and 40 DAA seed tissue. The quality of libraries was assessed using High Sensitivity DNA kit on Agilent 2100 Bioanalyzer (Agilent Technologies, California, USA). All the four cDNA libraries were sequenced in one complete run using the GS FLX Titanium series sequencing reagents and sequencer. Each of the cDNA libraries was tagged with unique RL MID (multiplex identifier) adaptors and mixed before emulsion PCR and sequencing.

### Assembly and annotation of 454 reads

The sequence data was obtained in Fastq format and various quality control steps were performed using the NGS QC tool kit (Patel and Jain, 2012). The sequence data obtained was deposited in the SRA database under the accession number SRX125162 (Run numbers SRR445088, SRR445089, SRR445090, and SRR445091 for 10 DAA, 20 DAA, 30 DAA, and 40 DAA seed tissue respectively). After passing through the quality controls, *de novo* assembly of filtered reads was done with Newbler v 2.3.5 software from Roche using default parameters. For the reference assembly, filtered reads were mapped onto the chickpea reference genome (Varshney et al., 2013) using gsMapper and assembled with Newbler v 2.3.5. Both assemblies were merged and clustered using TGICL (Pertea et al., 2003). To deduce their putative function, chickpea non redundant transcript data set was subjected to BLASTX analysis against the non-redundant protein database of UniProt Swiss-Prot (http://www.uniprot.org/downloads) and the hits with an *E*-value < 1*E*–05 were considered to be significant. The GOSlim terms for molecular function, biological process, and cellular component categories were assigned to the corresponding chickpea transcripts using in house Perl scripts. Distribution of transcripts into various biological pathways in KEGG was done through KAAS (KEGG Automatic Annotation Server; http://www.genome.jp/tools/kaas/). The transcripts were aligned to the sequences in the COG database (http://www.ncbi.nlm.nih.gov/COG) and categorized under different functions accordingly.

### Determination of transcript abundance and differential gene expression

Sequence reads from the four tissues, i.e., 10, 20, 30, and 40 DAA seed, were mapped onto the non-redundant chickpea transcripts using GS Reference Mapper (gsMapper, http://www.454.com/products/analysis-software/). Number of reads mapped were normalized and measured in terms of RPKM (reads per kilobase per million) in order to determine the transcript abundance (Mortazavi et al., 2008). Unigene expression levels were calculated as RPKM (A) = (10,00,000 × *C* × 1,000)/(*N* × *L*), where A refers to the expression of gene A, *C* to number of reads that uniquely aligned to gene A, *N* to total number of reads that uniquely aligned to all genes, and *L* to the length of gene A. The genes with normalized RPKM > 1 were considered to be expressed at a particular stage. The DEGseq package was used to determine differentially expressed genes at each stage (http://www.bioconductor.org/packages/release/bioc/html/DEGseq.html).

### Go term enrichment and biological pathway analysis

GO enrichment and pathway analysis was performed as described by Singh et al. (2013). Briefly, BLAST search was used to identify the best *Arabidopsis* hit corresponding to each chickpea transcript. GO enrichment of various sets of genes was performed using BiNGO tool (Maere et al., 2005). Transcript diversity and abundance was scrutinized with MapMan (Thimm et al., 2004) using the *M. truncatula* peptide database downloaded from MapMan (http://mapman.gabipd.org/web/guest/mapmanstore) as reference. Accordingly, chickpea seed transcripts were first compared (Blastx, evalue < 1E-05) against the *M. truncatula* peptide database downloaded from the ftp site of Phytozome v 9.1 (ftp://ftp.jgi-psf.org/pub/compgen/phytozome/v9.0/Mtruncatula/annotation/).

### Real time quantification

Gene specific primers were designed using PRIMER EXPRESS version 3.0 (PE Applied Biosystems, USA) with default parameters (Supplemental Table S2). First strand cDNA was synthesized by reverse transcription from 3 μg of total RNA in 20 μl of reaction volume using AccuScript High Fidelity 1st strand cDNA synthesis Kit (Agilent technologies, USA). 5X dilutions of all cDNA samples were used for Real time PCR analysis with 200 nM of each primer mixed with SYBR Green PCR master mix (Applied Biosystem, Life Technologies, USA) as per manufacturer's instructions. The reaction was carried out in 96-well optical reaction plates (Applied Biosystems, USA), using ABI Prism 7000 Sequence Detection System and software (PE Applied Biosystems, USA). To normalize the variance among samples, Elongation factor 1α (Forward: 5′-TCCACCACTTGGTCGTTTTG-3′, Reverse: 5′-CTTAATGACACCGACAGCAACAG-3′) (Garg et al., 2010) was used as endogenous control. Relative expression values were calculated after normalizing against the maximum expression value. The values presented are the mean of the three biological replicates, each with three technical replicates. The error bars indicate standard error. Correlation between log_2_ normalized values of qRT PCR and RPKM was plotted on MS Office Excel.

### Genomic DNA isolation and touchdown PCR

DNA was isolated from fresh, young leaf tissue of chickpea (ICCV2) using the CTAB method (Doyle and Doyle, 1987). The quality and final concentration was estimated by agarose gel electrophoresis using known concentration of uncut λ DNA as a standard. PCR amplification of genomic DNA was carried out in a 20 μl reaction volume in an Eppendorf Mastercycler ProS containing 30–50 ng of genomic DNA, PCR buffer (20 mM Tris–HCl, 50 mM KCl), 0.75 μM of each primer, 0.125 mM of each dNTP, 1.5 mM MgCl2 and 0.5 U of Taq DNA polymerase (Life Technology, India). The following touchdown amplification profile was used: (1) initial denaturation 94°C 3 min, (2) 18 cycles of 94°C 50 s, 60°C 50 s, decreasing annealing temperature 0.5°C/cycle, 72°C 50 s, (3) 20 cycles of 94°C 50 s, 50°C 50 s, 72°C 50 s, and (4) final extension 72°C 7 min. The amplification products were separated on 2% agarose gel (Lonza, Seakem), stained with ethidium bromide and analyzed using the gel documentation system Gel-Doc IT (UVP).

## Results

### Sequencing and assembly of chickpea seed transcriptome

cDNA libraries were prepared using the mRNA isolated from four stages of seed development i.e., 10 DAA, 20 DAA, 30 DAA and 40 DAA. Sequencing was carried out on the Roche 454 GS FLX Titanium platform and a total of about 1.38 million reads of Q20 quality were obtained (Table 1). The NGS QC ToolKit (Patel and Jain, 2012) was used for removing low quality reads, trimming adapter/primer sequences, trimming sequences containing homopolymers more than 7 bp in length and removing sequences less than 100 bp in length. The 1,129,347 high quality filtered reads obtained had an average length of 342 bp and covered a total of 332,667,201 bases. Amongst the four tissue types, the highest number of reads was recorded for 20 DAA seed tissue (420,329). All the high quality reads were *de novo* assembled using the gsAssembler software (Newbler v.2.3.5, from Roche, http://www.454.com/products/analysis-software/) and 86,905 unigenes were obtained. Reference based assembly was also done using the chickpea genome sequence (Varshney et al., 2013) as a reference which generated a total of 93, 911 unigenes. Both the *de novo* and reference assemblies were merged and clustered using TGICL (Pertea et al., 2003) to generate the final assembly containing 51,099 unigenes which was used for all further analyses (Table 2). The unigenes were used for BLASTX comparison with the peptide sequences of *C. arietinum* (Varshney et al., 2013) and showed 69.7% similarity. Similar comparison with the Non-redundant (Nr) protein database of NCBI (ftp://ftp.ncbi.nlm.nih.gov) showed that about 36,024 unigenes (70.5%) found a match while 15,075 unigenes (29.5%) found no hits in the database and hence can be assumed to be putative novel genes. Also, BLASTX comparison with protein sequences of *Arabidopsis, Oryza, Glycine, Lotus japonicus* and *Medicago* downloaded from Phytozome v 9.1 (http://www.phytozome.net/) showed that the chickpea seed transcriptome showed highest similarity with *G. max* (64.7%) and *M. truncatula* (60.6%) as compared to *L. japonicus* (59.8%), *A. thaliana* (57.6%) and *O. sativa* (53.7%).

**Table 2:** **Assembly statistics**.

**Type of assembly**	**N50 value**	**N50 Index value**	**Total no. of transcripts contigs+ singletons)**	**Avg. size of contig (bp)**	**Length of largest contig (bp)**	**No. of contigs 100–500 bp in size**	**No. of contig>500 bp in size**
*De novo*	441	22,089	86,905	437.6	6,483	72,490	14,415
Reference	541	22,065	93,911	415.96	12,845	64,743	29,168
*De novo* + reference (TGICL)	918	11,814	51,099	726.7	21,371	21,704	29,395

### Functional annotation

**Figure 1:**
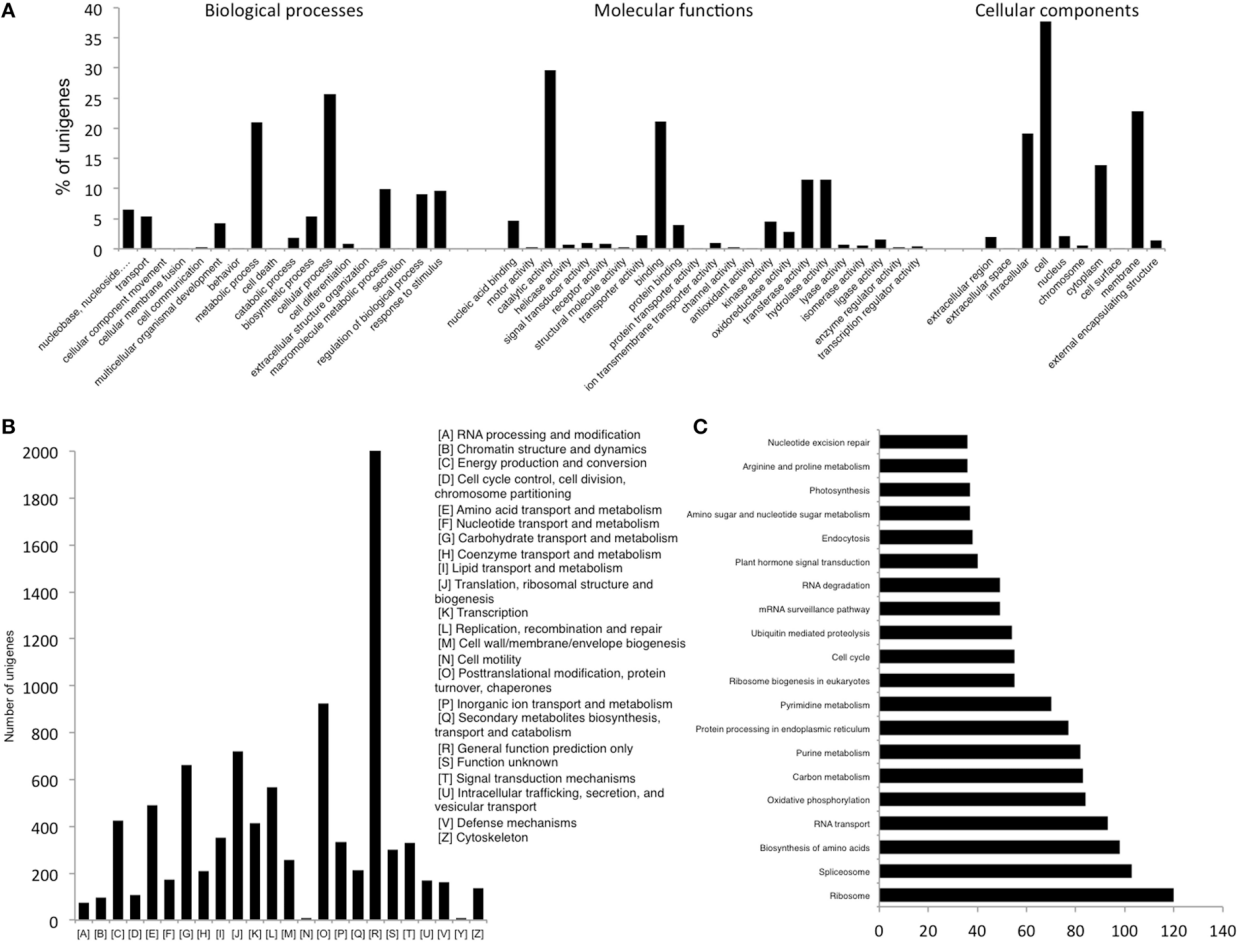
**(A)** GO Slim annotations for chickpea seed unigenes **(B)** Distribution of unigenes according to COG database **(C)** Distribution of unigenes into biological pathways using KEGG.

The assembly was annotated using Uniprot-SwissProt, COG and KEGG databases. The standalone version of NCBI Blast (version 2.2.25+) was downloaded from NCBI (ftp://ftp.ncbi.nlm.nih.gov/blast/executables/blast+/) and functional annotation of the transcripts was done by searching against the high quality non-redundant protein sequences in the UniProt Swiss-Prot database (http://www.uniprot.org) using BLASTX with a threshold *E*-value of < 1*E*–05. A total of 21,414 (41.9%) of the 51,099 unigenes could find a match in the Uniprot database, which were then annotated using in house Perl scripts. Accordingly, unigenes were assigned Gene Ontology (GO) terms and were grouped into the three main GO categories; Biological processes, Molecular function and Cellular components (Figure 1A). Majority of the unigenes were found to have a role in various Molecular functions followed by those related to Biological processes and Cellular components. Analysis of their further distribution into various sub categories revealed that the majority of the unigenes under Biological processes were involved in cellular (25.6%) and metabolic processes (21.05%). About 29.7% and 21.06% of unigenes under Molecular functions category were involved in catalytic activity and binding respectively while amongst the Cellular components, cell (37.74%) and membrane (22.8%) accounted for most of the unigenes (Figure 1A).

Another 9170 unigenes were annotated using the COG (Cluster of Orthologous Groups) database. Annotation according to these clusters classified majority of the unigenes (2033) under the “General function prediction only” category followed by “Post-translational modification, protein turnover, chaperones” (923) and “Translation, ribosomal structure and biogenesis” (721) (Figure 1B). Further, only 9.04% of the annotated unigenes could be distributed into 215 biological pathways enlisted in the KEGG (Kyoto Encyclopedia of Genes and Genomes) database (Figure 1C). Genes related to “Ribosome” were found to be most abundant in number (120) followed by those for “Spliceosome” (103) and “Biosynthesis of amino acids” (98).

### Transcriptional flux during progression of chickpea seed development

In order to elucidate the dynamics of seed development, we performed a number of analyses to determine the pattern of expression of unigenes across the four stages. The assembled set of 51,099 unigenes was used as the reference onto which raw reads from each stage were mapped to generate a putative expression profile for the transcripts. Unigenes with RPKM value > 1 in at least one of the seed tissues were considered to be trancriptionally expressed. We found 93.3% of the 51,099 unigenes to be expressing in at least one of the developmental stages and of these, 65.8% expressed at early stages of seed development (10 and 20 DAA) while 43.15% expressed at later stages (30 and 40 DAA). The unigenes were sorted into clusters using k Means/Median clustering function and Figure of Merit (FOM) calculation (Yeung et al., 2001) embedded in MeV v 4.9 to determine the optimal number of clusters to classify gene expression profiles, which resulted in 10 representative clusters. We selected five major clusters (K1–5) which displayed distinct patterns of expression during the seed development process (Supplemental Figure S1). Clusters K1 and K5 contained unigenes that showed a gradual increase and a gradual decrease toward later stages of development respectively. Seed storage proteins constituted majority of cluster K1 while in cluster K5 most of the metabolic enzymes and transcription regulators were seen to decrease as seed development progressed. Clusters K2, K3, and K4 contained unigenes that are highly expressed at 10, 20, and 30 DAA of seed development respectively. A number of heat shock proteins and Zn finger containing transcription factors were present in 10 DAA seeds while both 20 and 30 DAA seed tissue had abundance of various metabolic enzymes and a variety of transcription regulators (Supplemental Table S1). In order to validate the RNA-seq digital expression data, 20 unigenes were randomly selected, primers were designed (Supplemental Table S2) and qRT PCR was performed whereby we obtained a high correlation between the log normalized RPKM and RQ values (*r*^2^ = 0.69) (Supplemental Figure S2).

**Figure 2:**
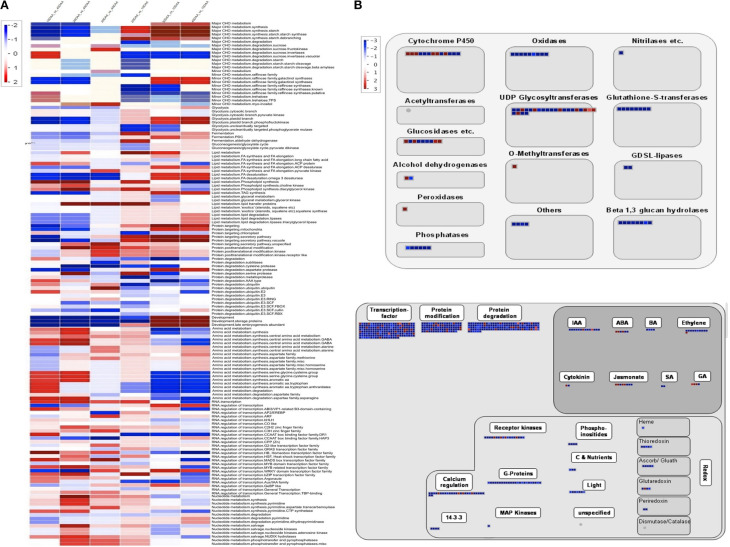
**(A)** PageMan based annotation of differentially expressed unigenes across different developmental stages. Scale on the left depicts level of expression; red being high and blue being low **(B)** MapMan pathways depicting regulation of seed maturation. (i) Large enzyme families differentially regulated at 30 and 40 DAA. (ii) Overview of regulation for 30 and 40 DAA seeds as compared to 10 DAA seed tissue. Scale on the left depicts level of expression; red being high and blue being low.

Further, DEGseq (Wang et al., 2010) was used to determine the differentially expressed unigenes across the four stages of seed development where only those unigenes which showed four-fold change in expression (*p*-value > 0.001, MARS method) were considered to be differentially expressed. Using this criterion, only 6.75% of the total set of unigenes was seen to be differentially expressed. In order to provide a more comprehensive idea of the pattern of gene expression, we generated two sets of data. The first set was derived using 10 DAA sample as control while in the second set, 40 DAA sample was used as control and the data was then subjected to a Bin-wise Wilcoxon test in PageMan (Figure 2A). Unigenes related to carbohydrate metabolism (major and minor CHO metabolism) were more highly expressed at later stages as compared to 10 DAA and included genes for starch synthases, debranching, starch cleavage, galactinol synthase etc. but genes for degradation of sucrose, starch and metabolism of raffinose and trehalose had higher expression in 10 DAA seed tissue as compared to 40 DAA. The processes of glycolysis, fermentation, gluconeogenesis, lipid metabolism, protein targeting and degradation and storage protein accumulation were observed to be generally upregulated in later stages compared to 10 DAA seed tissue. On the other hand, activities like amino acid metabolism, RNA: regulation of transcription, nucleotide metabolism were seen to be upregulated in earlier stages as compared to 40 DAA seed tissue. Although most DNA related processes were upregulated in 10 and 20 DAA seeds, some related to DNA synthesis (histones) were abundantly represented in 40 DAA seeds.

Seed maturation represents an interesting stage which may help understand the regulatory processes leading to quiescence. According to Setia and Malik (1985), increase in dry weight of chickpea seeds is more dramatic after 21 DAF (Days after Fertilization, synonymous to Days after Anthesis in case of chickpea which is a self-pollinating species) which continues upto 42 DAF. MapMan (Thimm et al., 2004) was used to analyse the large enzyme families represented in maturing chickpea seeds (30 and 40 DAA) (Figure 2B). We found that while most metabolic enzymes are downregulated in maturing seeds as compared to early stages, a few members of enzymes such as cytochrome P450, UDP-glycosyltransferases, peroxidases, alcohol dehydrogenases, O- methyltransferses are expressed preferentially in 30 and 40 DAA seed tissue. Almost all regulatory processes slow down as the seeds reach quiescence except for those related to hormones like ABA, GA, jasmonate, cytokinin and few members of IAA (Figure 2B).

### Identification of transcripts with preferential expression in seeds

**Figure 3:**
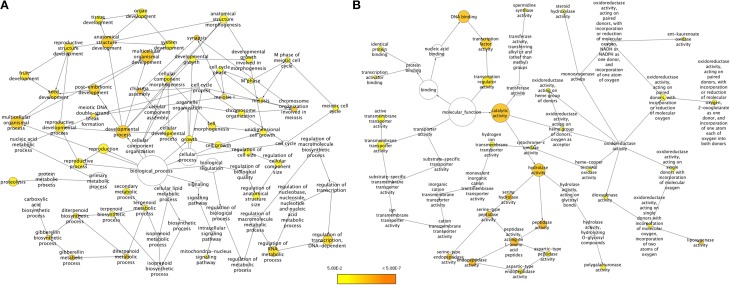
**GO enrichment of chickpea seed specific transcripts for (A) Biological processes and (B) Molecular functions**. Node size is proportional to the number of transcripts in each category and colors shaded according to the significance level.

We employed two approaches for identifying unigenes that are specific to chickpea seeds. In the first approach, unigenes obtained from the chickpea seeds were compared to the transcripts of both Desi (ICC4958) and Kabuli (ICCV2) varieties of chickpea available in the Chickpea Transcriptome Database (CTDB) (Garg et al., 2011b) which contain transcripts obtained from tissues of shoot, root, mature leaves, flowers and young pods but not from seeds. A comparison between the transcript data from seed and from the whole plant (as present in the CTDB, http://59.163.192.90:8080/ctdb/cgi-bin/downloadSeq.pl) revealed that of the 51,099 seed unigenes, 42,160 (82.5%) were common with the whole plant while 8,939 (17.5%) of seed unigenes did not find a match in the CTDB. These were therefore assumed to be putatively specific to seed tissue. GO enrichment of these chickpea seed specific unigenes was carried out using BiNGO based on homology with the *Arabidopsis* protein sequences (Figure 3). Under the Biological processes category, a clear enrichment of genes related to development processes especially, genes for seed development, embryonic and post embryonic development, fruit development, reproductive structure development and chiasma formation were seen to be highly represented (Figure 3A). We also found an over representation of categories like lipid metabolic process, terpenoid metabolic process, proteolysis, regulation of transcription, regulation of RNA metabolic process and gibberellin metabolic process. Categories like hydrolase activity, transcription factor activity and lipoxygenase activity were found to be overrepresented under Molecular functions (Figure 3B).

**Figure 4:**
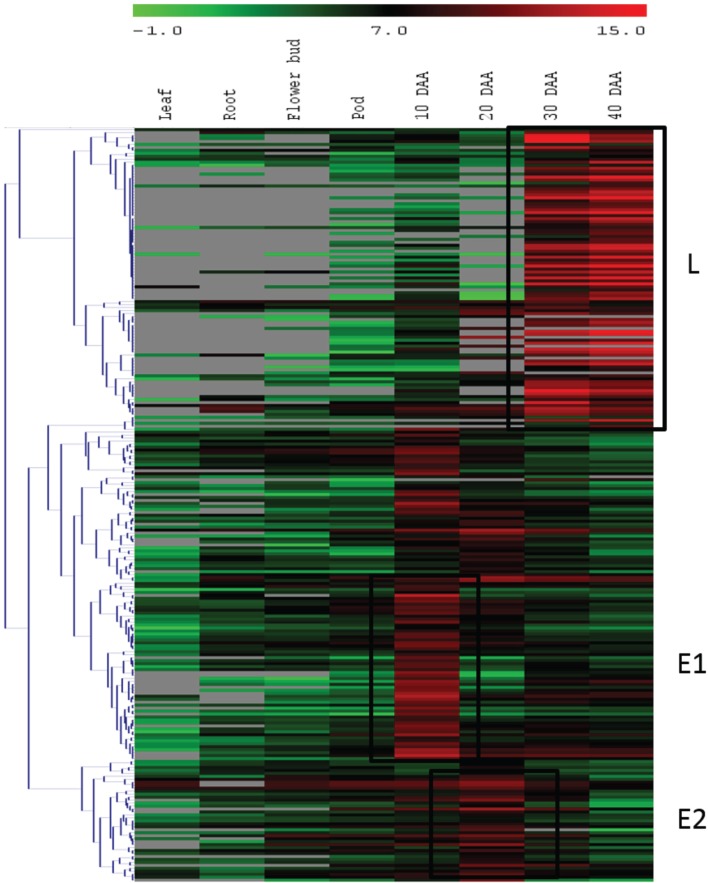
**Hierarchical clustering of unigenes with high expression**. Blocks E1 and E2 represent unigenes having higher expression at early stages and block L consists of unigenes expressed at higher levels at later stages of seed development.

**Figure 5:**
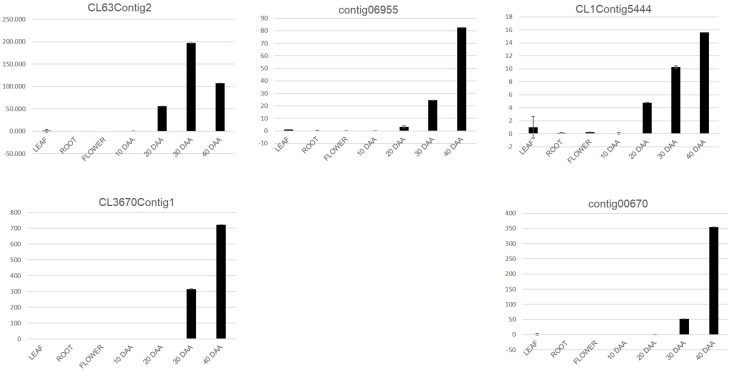
**qRT PCR analysis of putative seed specific unigenes**. The Y axis represents relative expression of genes as obtained by the 2^-ΔΔCt^ formula.

For the second approach, we mapped the transcript sequence reads generated from the four stages of developing seed (generated in this study) as well as the reads from the transcriptomes of leaf, root, flower bud and pod of chickpea reported by Garg et al. (2011b) onto the assembled unigenes from chickpea seed tissue. Log_2_ normalized RPKM values were calculated and subjected to Z-score analysis as described by Severin et al. (2010). The heat map was generated taking 1000 most highly expressing unigenes (calculated by taking sum of raw reads mapped onto each unigene) as well as the Z-scores above 2 in each seed tissue. Hierarchical clustering of these unigenes showed distinct blocks where unigenes with preferential expression in seed tissue could be found (Figure 4). We further analyzed these seed specific genes and found that almost all of the 10 DAA specific unigenes (Block E1; Figure 4) encoded heat shock proteins and chaperones. The 20 DAA seed had preferential expression of genes related to the proteasome machinery along with a variety of metabolic enzymes (Block E2; Figure 4). In later stages i.e., 30 and 40 DAA, while a sizeable portion of the highly expressing unigenes was constituted by storage proteins, a number of metabolic enzymes such as alcohol dehydrogenases, seed lipoxygenases and subtilisin proteases were also detected (Block L; Figure 4) (Supplemental Table S3). Five of the putative seed specific unigenes were analyzed using qRT PCR (Figure 5) in order to validate this approach.

### Transcription factors in chickpea seed development

**Figure 6:**
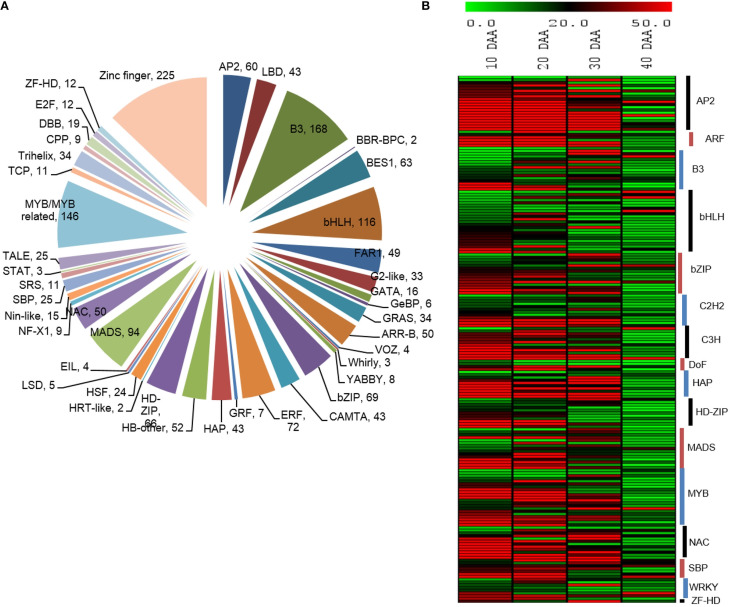
**(A)** Distribution of unigenes into TF families **(B)** Differential expression patterns of TFs in various stages of seed development.

Transcription factors play crucial roles in mediating transcriptional regulation. Therefore, we studied the expression dynamics of a number of transcripts which were identified to encode different transcription factors. Peptide sequences of different TFs from five different legumes (*Cajanus cajan, C. arietinum, G max, Lotus japonicus* and *M. truncatula*) were downloaded from Plant Transcription Factor Database (planttfdb.cbi.pku.edu.cn) and their HMM profiles were built using hmmbuild (HMMER 3.0, ftp://selab.janelia.org/pub/software/hmmer) and used for searching the chickpea seed transcriptome. We found 1,743 unigenes from the chickpea seed transcriptome to be transcription factors, which were further categorized into 54 transcription factor families of which the Zinc finger family was most abundant followed by B3, MYB, bHLH and MADS transcription factor families (Figure 6A). Based on the information obtained from previous reports (Verdier and Thompson, 2008; Agarwal et al., 2011), a number of transcription factors, which are known to be involved specifically in the process of seed development were analyzed for their pattern of digital expression in 10, 20, 30, and 40 DAA seed tissue (Figure 6B). We found that a majority of these transcription factors showed higher expression in early stages of seed development which decreased as the seed progressed to maturation. These included transcription factors belonging to AP2, ARF, HAP, bHLH, bZIP, NAC, and SBP families. However, a few members of MYB, bHLH, bZIP, C3H and WRKY had higher expression in 40 DAA seed tissue.

### Important secondary metabolites in chickpea seeds

Flavonoids and isoflavonoids are one of the most popular groups of secondary metabolites found in plants. Many legume seeds have been reported to be rich sources of these secondary metabolites (Prati et al., 2007; Wang et al., 2008; Heiras-Palazuelos et al., 2013). KEGG based annotation of the seed unigenes into various biological pathways revealed 12 enzymes involved in the flavonoid biosynthesis pathway and 5 in the isoflavonoid biosynthesis and these were represented by 29 unigenes in our transcriptome (Supplemental Figure S3).

**Figure 7:**
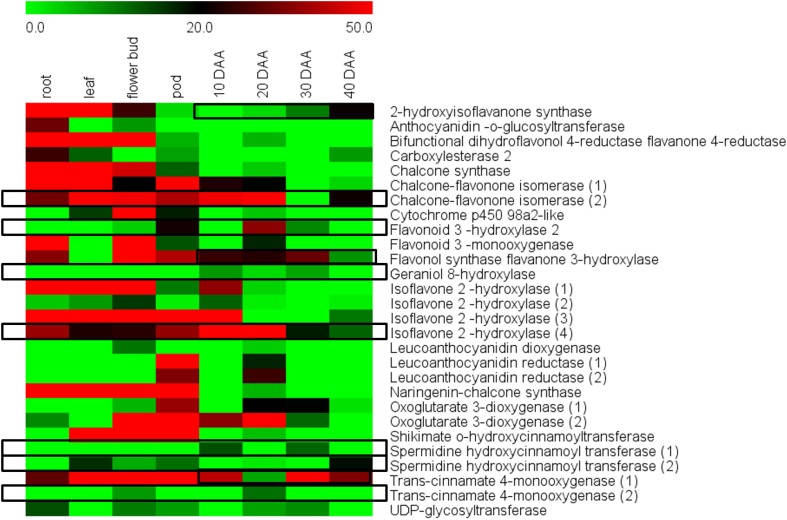
**Differential expression of genes for flavonoid and isoflavonoid biosynthesis in different chickpea tissues**.

An RPKM based analysis taking into account leaf, roots, flower bud and pods along with the four stages of seed development showed that most of the 29 unigenes had higher expression in vegetative tissues as compared to seeds (Figure 7, Supplemental Table S4). However, distinct exceptions to this trend were the enzymes isoflavone-2-hydroxylase (4), which is known to be involved in biosynthesis of hydroxygenistein and flavonoid 3 hydroxylase 2, which is known to be involved in biosynthesis of dihydroquercetin and quercetin. Isoflavone hydroxylase (4) was seen to have especially high expression at 10 DAA and its expression gradually declined toward 40 DAA while its expression remained comparatively low in non-seed tissues. The enzyme Flavonoid 3 hydroxylase was seen to have a distinctly higher expression in 20 DAA seed tissue while remaining low in all other tissues (Figure 6). Geraniol 8-hydroxylase and Spermidine hydroxycinnamoyl transferase (1) also showed preferential expression in 10 and 30 DAA seed tissue while Chalcone-flavonone isomerase (2) and Trans-cinnamate 4-monooxygenase (2) showed higher expression in 20 DAA seed tissue as compared to other samples (Figure 6). Although differential expression analysis showed that most secondary metabolic activities are suppressed in later stages, some enzymes like Trans-cinnamate 4-monooxygenase (1), 2-hydroxyisoflavanone synthase, Spermidine hydroxycinnamoyl transferase (2) and Flavonol synthase showed higher expression in 30 and 40 DAA seed tissue as compared to 10 and 20 DAA seeds.

### SSR mining and validation

**Figure 8:**
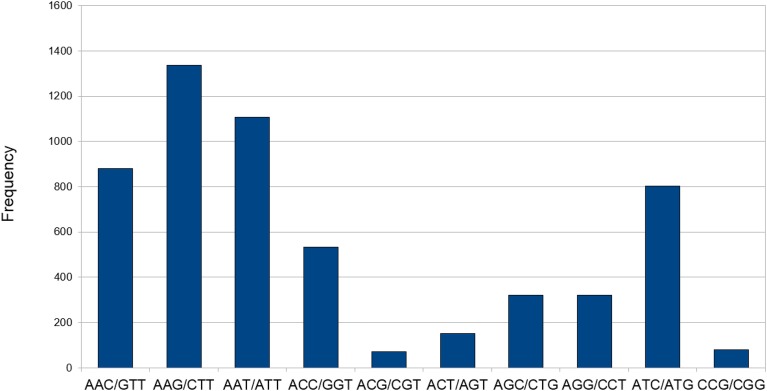
**Abundance of different types of trinucleotide repeat containing SSRs**.

Transcriptome data is very widely used as a source to develop molecular markers especially the Simple Sequence Repeats (SSRs). Therefore, in order to identify SSRs (dinucleotide to hexanucleotide repeats) in transcripts of developing seeds of chickpea, we mined the transcripts generated in this study using the MISA Perl script. Mononucleotide repeats were not considered as we trimmed sequence reads with homopolymers of more than seven bases as a part of quality control. A total of 12,606 SSRs could be identified in 9305 (18.2%) transcripts of chickpea (Table 3). The average frequency of SSRs was found to be one SSR per 2.94 kb of the chickpea seed transcriptome sequence. Furthermore, 2249 transcripts contained more than one SSR, and 841 SSRs were present in compound formation. The largest fraction of SSRs identified were trinucleotides (44.46%) followed by tetranucleotides (25.82%). Among the trinucleotide repeat containing SSRs identified, AAG/CTT accounted for majority (24%) of the repeats followed by AAT/ATT which accounted for 20% of trinucleotide repeats (Figure 8). Moreover, attempts were made to identify the novel SSRs out of the set here. These SSRs (including 200 bp upstream and downstream flanking sequences), were compared to the 48,328 SSR sequences reported from whole chickpea genome (Varshney et al., 2013). We found that only 1557 chickpea seed SSRs found matches with those from the chickpea genome. So, it can be assumed that the remaining 11,049 SSRs from chickpea seed are novel in chickpea. Forward and reverse primers were designed for 48 SSRs (Supplemental Table S2) and 43 of these (89.58%) were successfully amplified from genomic DNA of *C. arietinum* (ICCV2) (Supplemental Figure S4).

**Table 3:** **Numbers and distribution of SSRs in chickpea seed tissue**.

Total number of sequences examined	51,099
Total size of examined sequences (bp)	37,134,000
Total number of identified SSRs	12,606
Number of SSR containing sequences	9,305
Number of sequences containing more than 1 SSR	2,249
Number of SSRs present in compound formation	841
**DISTRIBUTION OF SSRS ACROSS DIFFERENT REPEAT TYPE CLASSES**	
**Unit size**	**Number of SSRs**
2 (dinucleotides)	1,624
3 (trinucleotides)	5,604
4 (tetranucleotides)	3,255
5 (pentanucleotides)	1,075
6 (hexanucleotides)	1,048

## Discussion

### RNA seq is a useful method for resource generation

Analysis of transcriptomes is crucial as it not only helps in large scale identification of mRNA but also provides insights into the molecular basis of genes involved in developmental processes. Seed development is an important process in plants and understanding the developmental dynamics, especially in plants of economic importance where seeds serve as a source of food and feed, may greatly help in developing crop improvement strategies. The ICCV2 cultivar of Kabuli chickpea was selected for analysis as it has gained distinction for being the first extra-short-duration (85–90 days) Kabuli variety with *Fusarium* wilt resistance and heat tolerance which can also escape terminal drought due to early maturation. In addition to this, the seeds of this cultivar are valued due to their higher protein content (23.4% as compared to 19.4% in Desi varieties) and better quality traits like biological value, true digestibility, net protein utilization etc., (ICRISAT, 1990).

Described by Margulies et al. (2005), the first NGS platform to integrate pyrosequencing using their PTP device was commercialized by Roche/454. Unlike platforms that produce shorter read lengths, the Roche/454 platform does not require the run time to be doubled for the sequencing of mate-pair templates. In addition, the long reads produced by the 454 platform are especially useful for *de novo* assembly of non-model transcriptomes (Vera et al., 2008). We have used both the *de novo* as well as the reference based assemblies of the chickpea seed transcriptome to generate a more comprehensive and inclusive set of unigenes that provide an unbiased representation of the four distinct stages of chickpea seed development. The assembly statistics revealed that the merged assembly had better characteristics, such as higher N50 value, average contig length, total number of contigs, longest contig length etc. which serve as a yardstick to assess the quality of an assembly.

Transcriptomes serve as a source of valuable information, especially, discovery of genes and markers (Garg et al., 2011a). Most of the conventional methods fall short in terms of coverage as well as handling of data. Out of the unigenes generated in this study, 18.2% were found to be containing SSRs with an average frequency of 1 SSR per 2.94 Kb of sequence, which is higher than that of the rest of the chickpea plant (Garg et al., 2011a). Further analysis revealed that of the total set of mined SSRs, about 87% were novel and moreover about 90% were validated in genomic DNA of chickpea thereby suggesting that the SSRs generated in this study had considerable utility for marker-assisted selection in chickpea breeding programs.

### Chickpea seed development is a dynamic process

The overall process of chickpea seed development was seen to be similar to that of other dicot seeds whereby the cell division phase is followed by cell expansion and then by maturation. Nevertheless, a more in depth analysis revealed a number of processes that may be more specific to seed development as compared to the rest of the plant. Gene Ontology based annotation revealed enrichment of categories like catalytic activity, binding, cellular and metabolic processes and cellular components like cell and membrane which clearly indicate that the process of seed development is genetically programmed and therefore may be correlated to increased metabolic and catalytic activities in the cell and membranes (Borisjuk et al., 2004). In contrast, the study of the transcriptomes from 5 other tissues of chickpea (Garg et al., 2011a) revealed the highest number of transcripts to have a function in the chloroplast and plasma membrane, as well as in processes like protein metabolism and response to various stimuli and stress. It was very clear that expression of most of the unigenes occured at early stages suggesting a gradual decrease in transcriptome dynamics as the seed progressed toward desiccation and attaining quiescence. Moreover, the early stages were marked by increased metabolic activity involving high protein turnover (synthesis and degradation) whereas the later stages were characterized by protein storage and nutrient reservoir activities. It was also evident that the majority of the unigenes expressed across all stages of seed development with only 6.75% of unigenes having differential expression in different stages of seed development. This was in agreement with earlier studies of soybean (Severin et al., 2010) and *Arabidopsis* (Le et al., 2010) which had suggested that most genes involved in various seed functions are shared by all stages of seed development although there might be significant changes in their transcriptional activities. Nevertheless, each developmental stage may have a very small gene set specific to that stage (Sreenivasulu and Wobus, 2013). Hierarchical clustering of the most highly expressing genes revealed a remarkably high expression of heat shock proteins in 10 DAA tissue suggesting the formation of nascent proteins since the general role of heat shock proteins is to act as molecular chaperones regulating the folding and accumulation of proteins as well as localization and degradation in all plant and animal species (Feder and Hofmann, 1999; Schulze-Lefert, 2004; Panaretou and Zhai, 2008; Hu et al., 2009; Gupta et al., 2010) especially the Hsp70, which functions as a chaperone for newly synthesized proteins to prevent their accumulation as aggregates by folding in a proper way during transfer to their final location (Sung et al., 2001; Su and Li, 2008). Similarly, at later stages of development (30 and 40 DAA), there was an abundance of genes related to nutrient reservoir activity, thus emphasizing the process of storage protein accumulation. But distinct expression of any particular category of transcripts was not seen in 20 DAA tissue although this stage displayed a high expression of most genes such as those belonging to categories like catalytic activity, metabolic process, carbohydrate metabolic process etc. This reiterates the hypothesis that the process of seed development is a time of high metabolic activity where early stages are associated with nascent protein generation as well as degradation while most of the storage protein accumulation occurs at later stages.

### Genes regulating seed development

Comparison with the known chickpea transcripts from 5 tissues in the CTDB (Garg et al., 2011b) helped in identifying putative seed specific transcripts among which, presence of transcripts related to seed storage proteins and lipoxygenases along with enrichment of genes for seed, embryo, fruit development transcription activity, lipid metabolism and terpenoid metabolism not only further validated the seed transcriptome but also pointed to the importance of these gene groups in the process of seed development. Lipoxygenases are an important group of enzymes which catalyze the oxidation of polyunsaturated fatty acids such as linoleic (18:2) and α-linolenic (18:3) to produce unsaturated fatty acid hydroperoxides (Liavonchanka and Feussner, 2006) while alcohol dehydrogenases encode glycolytic enzymes. Preferential expression of these enzymes at later stages of seed development along with seed storage proteins add value to the nutritive quality of chickpea seeds.

Transcription factors are crucial regulatory proteins which mediate transcriptional regulation. They are known to be involved in almost every key process that occurs during plant development. Since seed development is such a dynamic process, it was imperative that we study the TFs involved. We found that majority of the TFs involved in seed developmental processes were highly expressed in early stages i.e. in 10 and 20 DAA seed tissue. Most of the well-known TFs known to be involved in seed development were also found in our transcriptome thereby reiterating the validity of our study. Almost all of the AP2 and HAP TFs were seen to be expressed at early stages while a few members of WRKY and C3H Zn finger containing TFs had higher expression at later stages. This reinforces the observation that gene activity in seeds decreases as they reach maturity (Sreenivasulu and Wobus, 2013). Such biased pattern of expression favoring early stages of chickpea seed development also suggests that most TFs are involved in processes like protein turnover, synthesis of storage compounds and general metabolism since these processes are abundant during early seed development. This also suggests that a very small proportion of these regulatory proteins are involved in the process of achieving dormancy since most gene activity ceases during later stages of chickpea seed development. Further studies related to this small group of TFs can lead to deciphering of many processes related to seed dormancy.

### Chickpea seeds can be a source of antioxidants

During this study, we recurrently encountered a number of genes related to secondary metabolic processes. The potent antioxidant properties of flavonoids like genistein and quercetin have been well investigated (Borras et al., 2006; Boots et al., 2008; Zhang et al., 2010). Enzymes involved in biosynthesis of these flavonoids had distinctly higher expression in 10 and 20 DAA seed tissue suggesting that genistein and quercetin accumulate preferentially in chickpea seeds as compared to rest of the plant. We also observed that although most gene activity slows down as the seed nears quiescence, some enzymes related to flavonoid biosynthesis are upregulated in the later stages. This suggests that flavonoid biosynthesis may be closely associated with chickpea seed maturation. Hence, chickpea seeds can be considered as a good source of antioxidants along with proteins and carbohydrates.

In conclusion, studying molecular mechanisms underlying plant development are usually difficult but extremely important. Although molecular aspects of seed development have been investigated in model plants such as *Arabidopsis* and *Medicago*, studying the process in important crop legumes such as chickpea is necessary to unravel any unique processes regulating chickpea seed development. This deep transcriptome sequencing analysis revealed that the overall pattern of seed development was largely common in chickpea and other dicot seeds. However, preferential expression of certain genes/families may be responsible for the unique nutritive values of chickpea seeds. Hence this study would serve as a foundation for characterization of candidate genes which would not only provide novel insights into understanding seed development but also provide resources for improved chickpea breeding.

### Conflict of interest statement

The authors declare that the research was conducted in the absence of any commercial or financial relationships that could be construed as a potential conflict of interest.

## Footnotes

^1^Available online at: http://faostat.fao.org
