# Supplementary figures and images for: Global transcriptome analysis of developing chickpea (Cicer arietinum L.) seeds
Source: Front Plant Sci. 2014 Dec 16;5:698. doi: 10.3389/fpls.2014.00698 (PMC4267183; doi:10.3389/fpls.2014.00698)

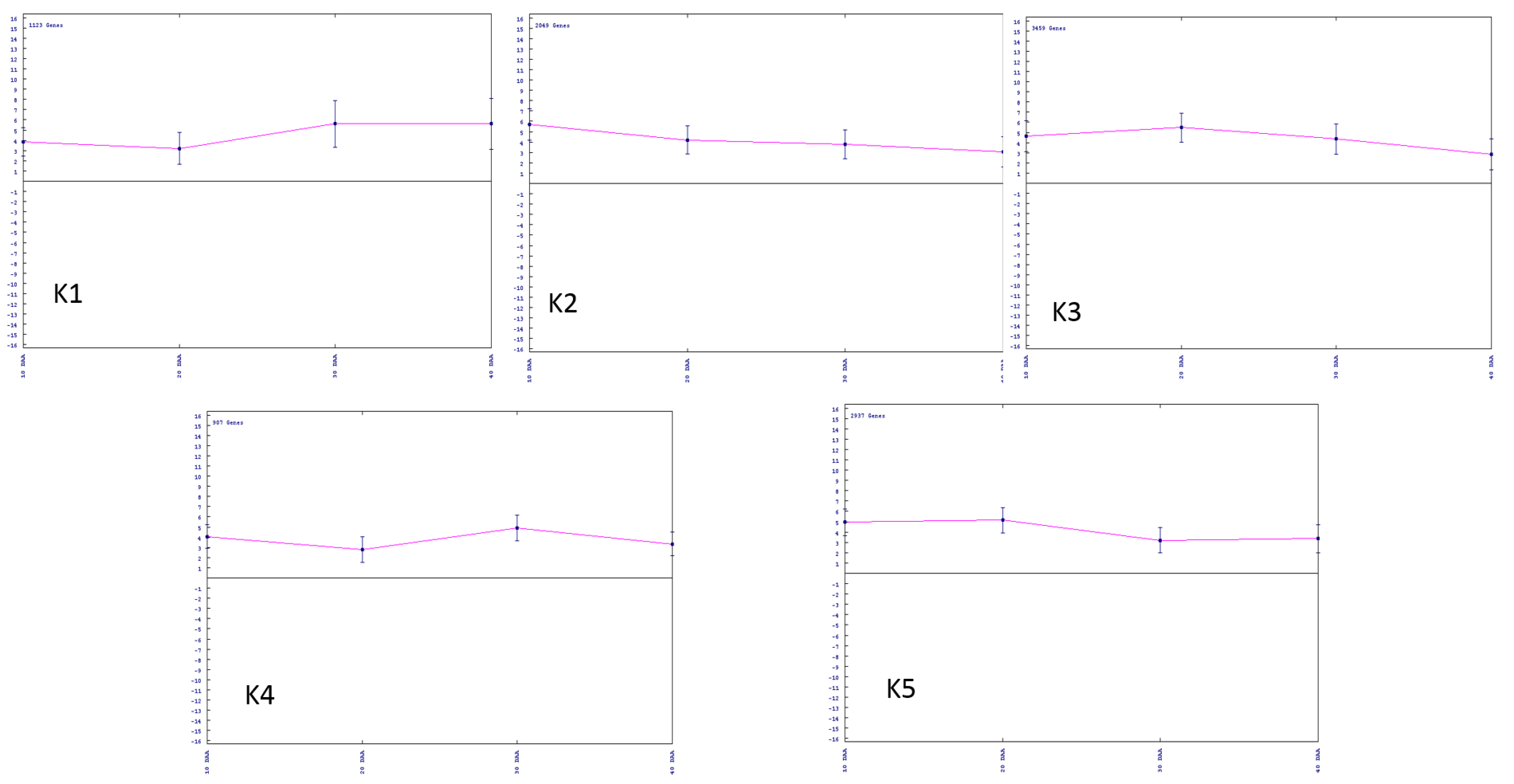

Supplement: Supplementary file 5 [file Image1.TIFF]

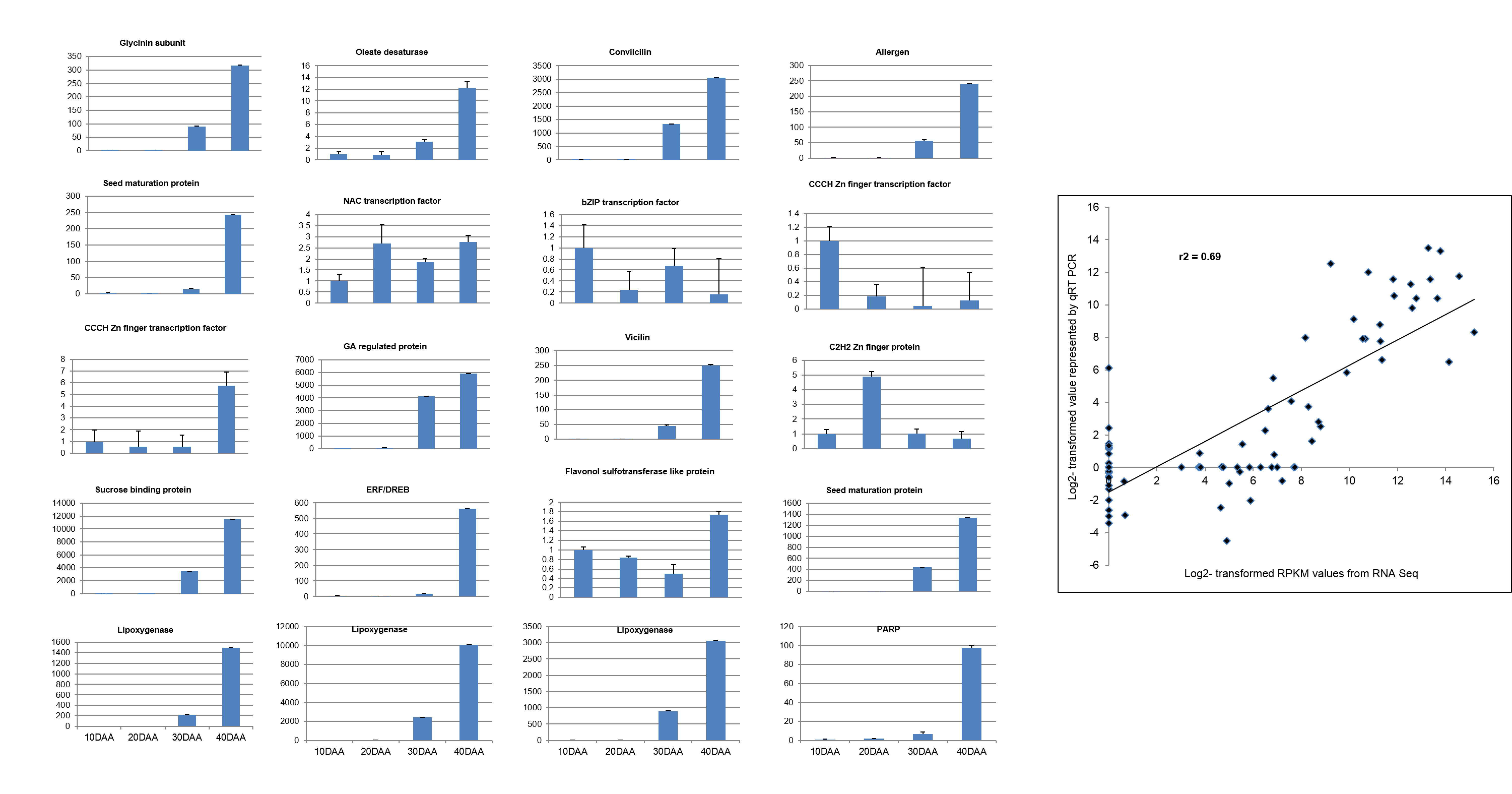

Supplement: Supplementary file 6 [file Image2.JPEG]

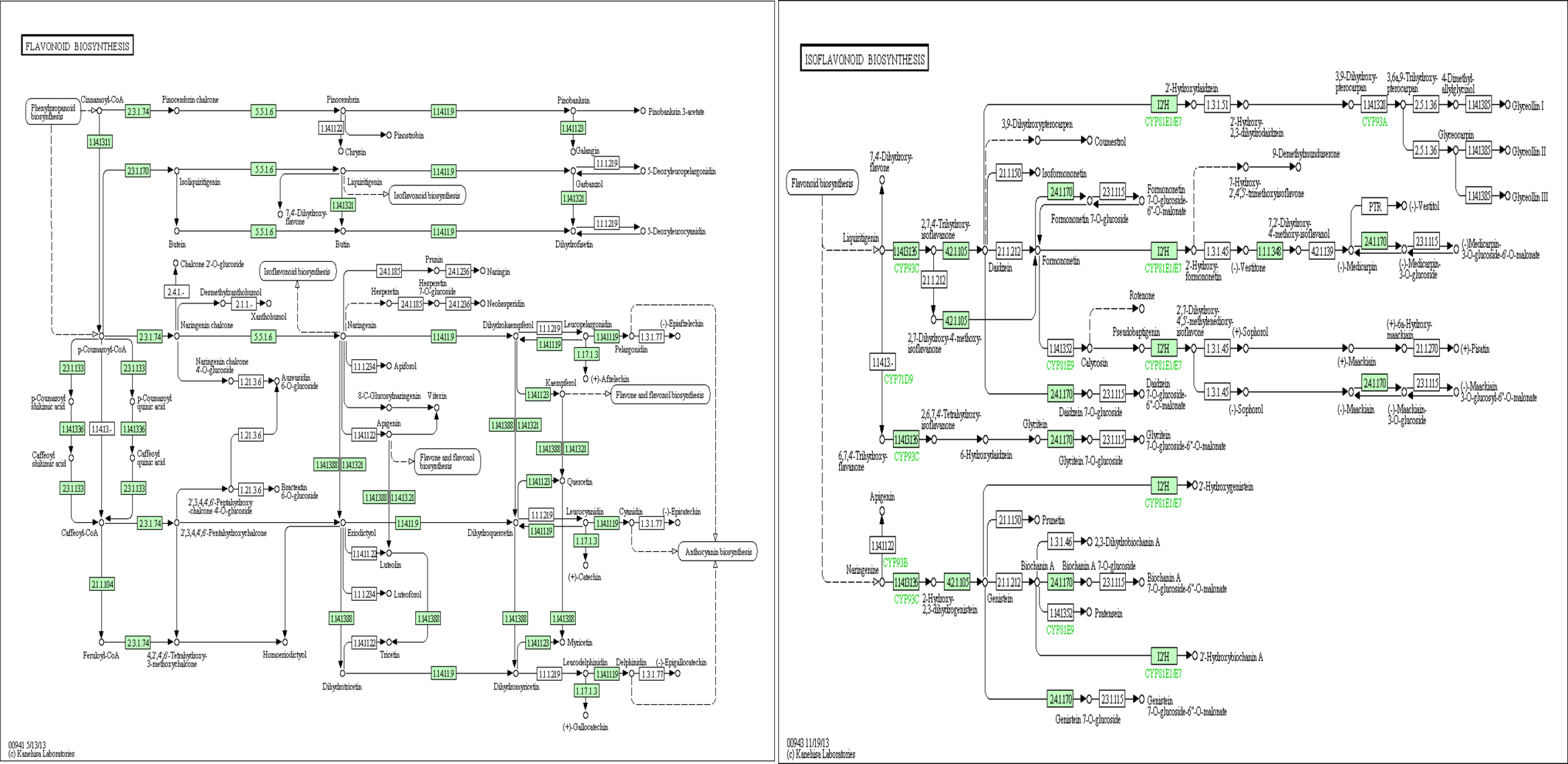

Supplement: Supplementary file 7 [file Image3.TIFF]

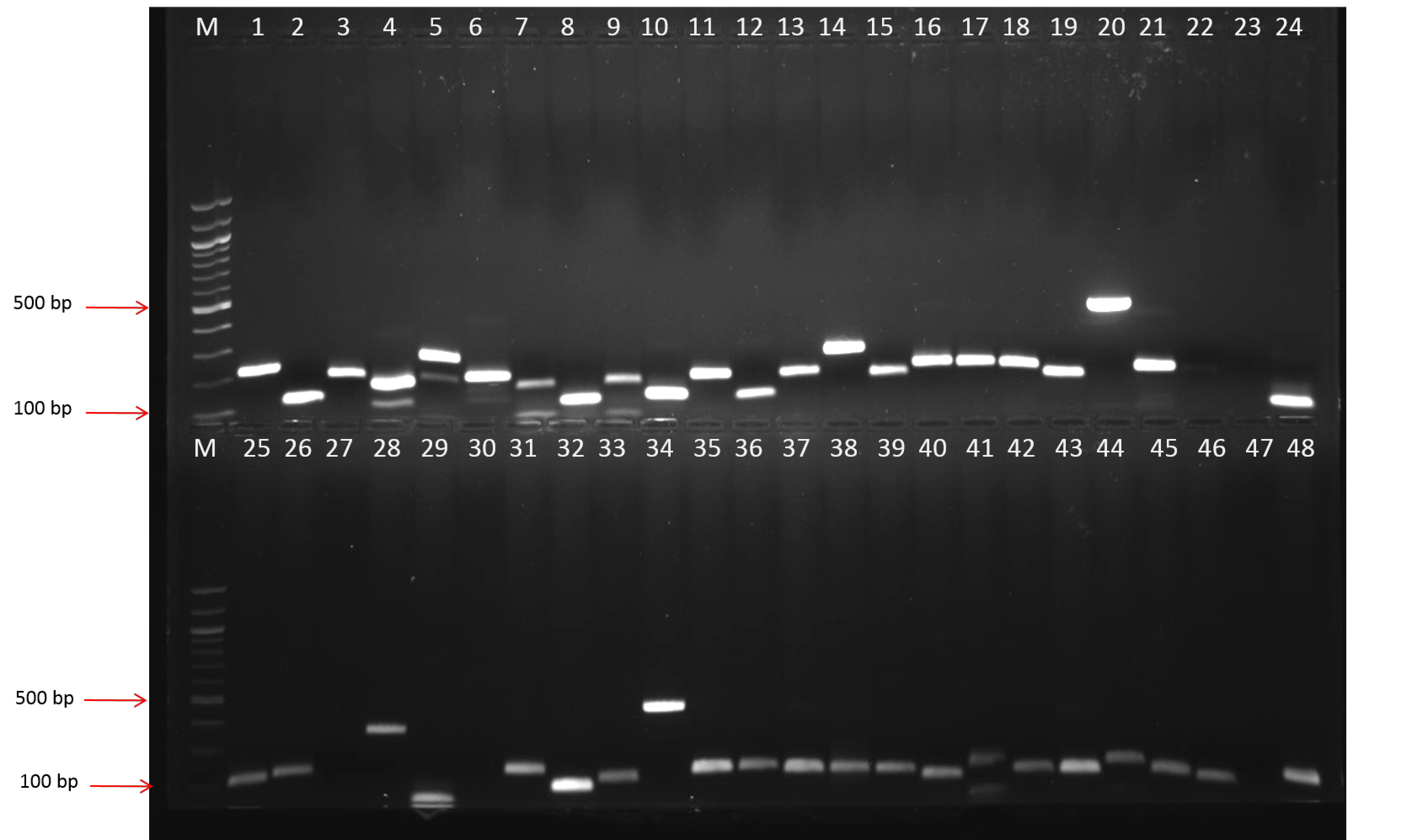

Supplement: Supplementary file 8 [file Image4.TIFF]
